# Porcine Dental Epithelial Cells Differentiated in a Cell Sheet Constructed by Magnetic Nanotechnology

**DOI:** 10.3390/nano7100322

**Published:** 2017-10-13

**Authors:** Wataru Koto, Yoshinori Shinohara, Kazuyuki Kitamura, Takanori Wachi, Seicho Makihira, Kiyoshi Koyano

**Affiliations:** Section of Fixed Prosthodontics, Department of Oral Rehabilitation, Faculty of Dental Science, Kyushu University, Fukuoka 812-8582, Japan; k.wataru@dent.kyushu-u.ac.jp (W.K.); k.kitamura@dent.kyushu-u.ac.jp (K.K.); wachi@dent.kyushu-u.ac.jp (T.W.); makihira@dent.kyushu-u.ac.jp (S.M.); koyano@dent.kyushu-u.ac.jp (K.K.)

**Keywords:** magnetic nanoparticles, nanotechnology, cell sheet, odontogenic cells, epithelial-mesenchymal interactions, dental enamel regeneration

## Abstract

Magnetic nanoparticles (MNPs) are widely used in medical examinations, treatments, and basic research, including magnetic resonance imaging, drug delivery systems, and tissue engineering. In this study, MNPs with magnetic force were applied to tissue engineering for dental enamel regeneration. The internalization of MNPs into the odontogenic cells was observed by transmission electron microscopy. A combined cell sheet consisting of dental epithelial cells (DECs) and dental mesenchymal cells (DMCs) (CC sheet) was constructed using magnetic force-based tissue engineering technology. The result of the iron staining indicated that MNPs were distributed ubiquitously over the CC sheet. mRNA expression of enamel differentiation and basement membrane markers was examined in the CC sheet. Immunostaining showed Collagen IV expression at the border region between DEC and DMC layers in the CC sheet. These results revealed that epithelial–mesenchymal interactions between DEC and DMC layers were caused by bringing DECs close to DMCs mechanically by magnetic force. Our study suggests that the microenvironment in the CC sheet might be similar to that during the developmental stage of a tooth bud. In conclusion, a CC sheet employing MNPs could be developed as a novel and unique graft for artificially regenerating dental enamel.

## 1. Introduction

Magnetic nanoparticles (MNPs) are widely used in medical treatments and clinical and basic research because of their unique features, such as superparamagnetic properties, biocompatibility, and non-toxicity. MNPs are used as contrast media and have been extensively used in the field of magnetic resonance imaging due to their better imaging resolution and sensitivity [1,2]. Since the technology of coating molecules and antibodies on the surface of MNPs was well established, MNPs have been applied in drug delivery systems and hyperthermia in cancer therapy [3,4,5,6,7,8]. MNPs can also be used to decorate biological nanofibers [9]. These nanofibers decorated with MNPs can be induced to form biological nanofiber assemblies on the centimeter scale by applying magnetic force. These long-range-ordered assemblies can be used as scaffolds in tissue engineering to encourage the adhesion, proliferation, and differentiation of various cells [9].

In the field of regenerative medicine, cell sheets created by magnetic force-based tissue engineering technology (Mag-TE) have been applied to the regeneration of many organs, including skin, bone, liver, and heart. Such cell sheets prepared by Mag-TE are reported to be functional and have favorable structures [10,11,12,13,14]. It is reported that multilayered keratinocyte sheets can be constructed by using Mag-TE system and be harvested without enzymatic treatment [10]. The transplantation of mesenchymal stem cell (MSC) sheets constructed by the Mag-TE system induced bone formation in bone defect areas in the crania of nude rats, and it is suggested that these MSC sheets are useful for bone tissue engineering [11]. By using the Mag-TE system, human aortic endothelial cells (HAECs) adhered to form a layered construct with tight and close contact on rat hepatocyte monolayers [12]. In this co-cultured construct, albumin secretion by hepatocytes was significantly enhanced compared with that in homotypic cultures of hepatocytes or heterotypic co-cultures of hepatocytes and HAECs without using the Mag-TE system. In heart revision, the presence of gap junctions and electrical connections were found within the cardiomyocytes sheets using the Mag-TE system [13]. In addition, it was demonstrated that human iPS cell-derived fetal liver kinase-1 positive cell sheets accelerated revascularization of ischemic hindlimbs in nude mice [14]. These several reports suggest that MNPs and the Mag-TE system are useful for regenerative medicine.

There has been great interest in the regeneration of tooth and periodontal tissue. Although attempts at reconstruction of dentin, periodontal ligaments, and alveolar bone have been relatively successful, regeneration of dental enamel (DE) remains difficult because of problems due to its specific developmental process [15,16]. It has been reported that DE is formed and matured by epithelial–mesenchymal interactions. Similar to the development of tooth germ, the establishment of such constant epithelial–mesenchymal interactions depends heavily on the positional relationship between dental epithelial cells (DECs) and dental mesenchymal cells (DMCs) [15,17]. However, no method has been developed to address the issue of cell position. Therefore, we attempted to solve this problem by focusing on MNPs, materials that can be used to control the arrangement of DECs and DMCs [18], and hypothesized that we could imitate the microenvironment in the developmental stage of a tooth bud in vitro.

In this study, we investigated the effects of MNPs and exposure to magnetic force on DECs and DMCs obtained from porcine tooth germ. We constructed a combined cell sheet consisting of DECs and DMCs (CC sheet) using the Mag-TE system, and examined the expression of differentiation and basement membrane markers in this cell sheet.

## 2. Results

### 2.1. Internalization of MNPs in Odontogenic Cells 

**Figure 1:**
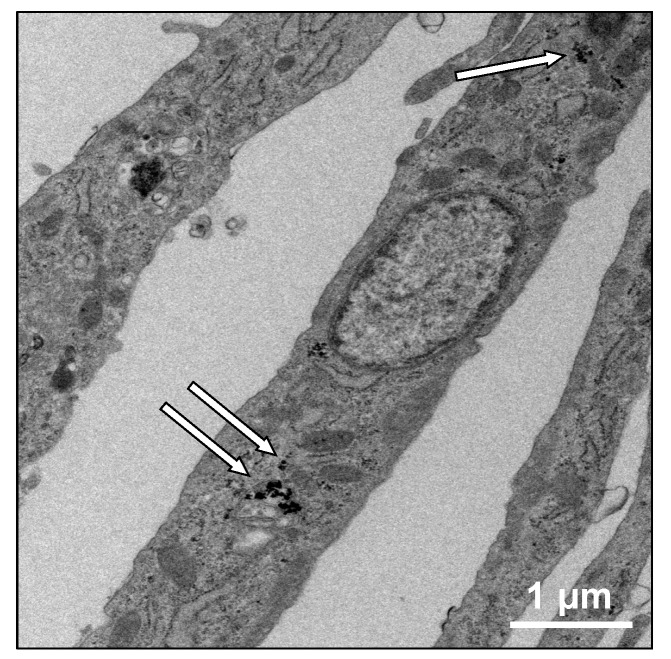
Transmission electron microscopy (TEM) image of odontogenic cells treated with 100 pg-magnetite/cell of magnetic nanoparticles (MNPs) (×5000). White arrows indicate intracellular MNPs. Scale bar = 1 μm.

Internalization of MNPs in odontogenic cells was evident from transmission electron microscopy (TEM) microphotographs (Figure 1). The presence of MNPs in the cytoplasm was confirmed. The diameters of MNPs were also measured on TEM images at higher magnification and the average was approximately 20 nm (data not shown).

### 2.2. Cytotoxicity of MNPs in DECs and DMCs

**Figure 2:**
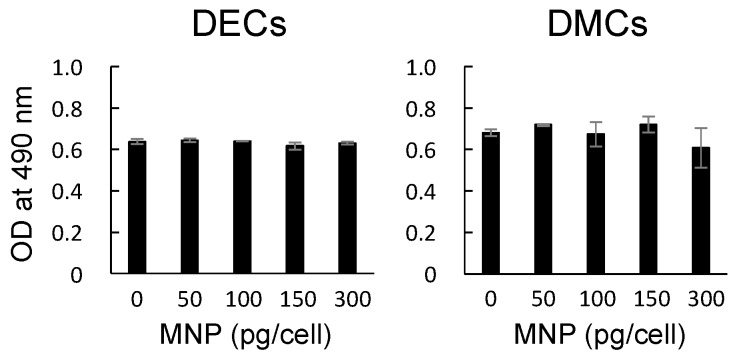
Cytotoxic effects of MNPs on dental epithelial cells (DECs) and dental mesenchymal cells (DMCs) assessed by MTS assays. MNPs at 0, 50, 100, 150, or 300 pg-magnetite/cell were added to the cells at confluency. After the cells were maintained for 24 h in the presence or absence of MNPs, the MTS assay was performed. *p* > 0.05, ANOVA.

The cytotoxic effects of MNPs on DECs and DMCs were assessed by MTS assays. MNPs at 0, 50, 100, 150, or 300 pg-magnetite/cell were added to DECs and DMCs at confluency. There were no significant changes in the absorbances at all concentrations for both DECs and DMCs (*p* > 0.05, one-way analysis of variance (ANOVA) with Tukey’s multiple range test) (Figure 2). 

### 2.3. Effects of MNPs and/or Magnetic Force on mRNA Expression of Enamel Matrix Genes in DECs

**Figure 3:**
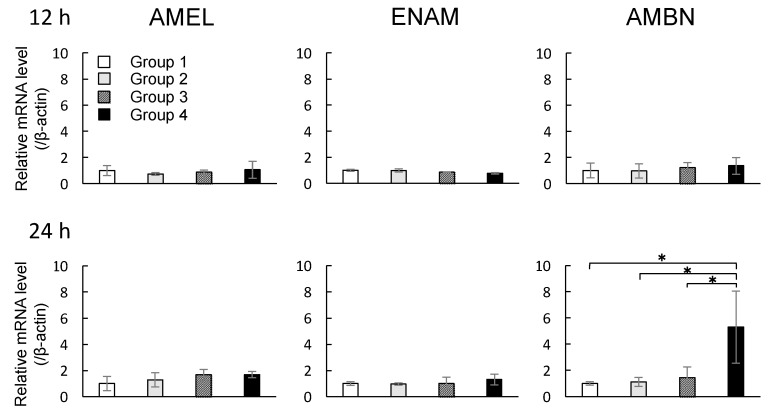
Effects of MNPs and/or magnetic force on the expression levels of *AMEL*, *ENAM*, and *AMBN* in cultured DECs examined by real-time reverse transcriptase polymerase chain reaction (RT-PCR). Real-time RT-PCR data were normalized to the expression levels of *β-actin* mRNA. Independent experiments were repeated twice. Data represent the mean ± standard deviation (SD) of triplicate samples. * *p* < 0.05, ANOVA.

The mRNA expression profiles of *Amelogenin* (*AMEL*), *Enamelin* (*ENAM*), and *Ameloblastin* (*AMBN*) in DECs were investigated after the cells were exposed to MNPs and/or magnetic force for 12 and 24 h. Depending on the presence of MNPs or magnetic force, experimental groups were classified into four subgroups as shown in Table 1. There were no significant changes in the levels of all mRNAs in groups 1–4 when the magnetic force was applied for 12 h (*p* > 0.05, ANOVA). Group 4 with 24 h of exposure to magnetic force showed significant increases in the mRNA expression of *AMBN* compared with groups 1–3 (* *p* < 0.05, ANOVA), although there were no significant changes in the levels of *AMEL* or *ENAM* mRNAs (*p* > 0.05, ANOVA) (Figure 3). 

### 2.4. Effects of MNPs and/or Magnetic Force on the mRNA Expression of Dentin-Related Genes in DMCs

**Figure 4:**
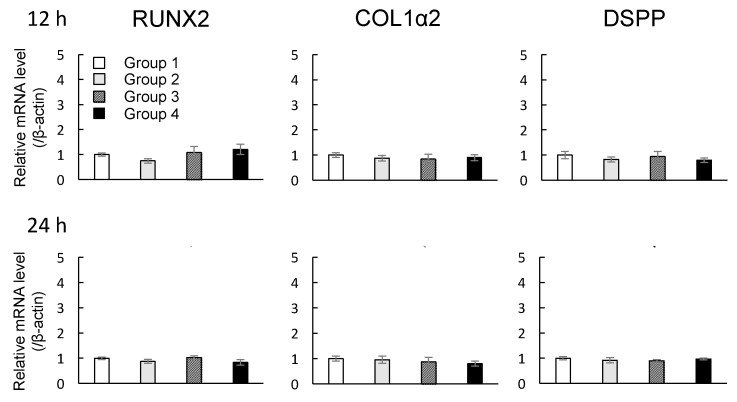
Effects of MNPs and/or magnetic force on the expression levels of *RUNX2, COL1α2*, and *DSPP* in cultured DMCs examined by real-time RT-PCR. Real-time RT-PCR data were normalized to the expression levels of *β-actin* mRNA. Independent experiments were repeated twice. Data represent the mean ± SD of triplicate samples. *p* > 0.05, ANOVA.

The mRNA expression profiles of *Runt-related transcription factor 2* (*RUNX2*), *CollagenIα2* (*COL1α2*), and *Dentin sialophosphoprotein* (*DSPP*) in DMCs were investigated after the cells were exposed to MNPs and/or magnetic force for 12 and 24 h. Depending on the presence of MNPs or magnetic force, experimental groups were classified into four subgroups as shown Table 1. There were no significant changes in the levels of *RUNX2*, *COL1α2*, or *DSPP* mRNAs in groups 1–4 when the magnetic force was applied for 12 or 24 h (*p* > 0.05, ANOVA) (Figure 4).

### 2.5. Construction of DEC and DMC Sheets Using the Mag-TE System

**Figure 5:**
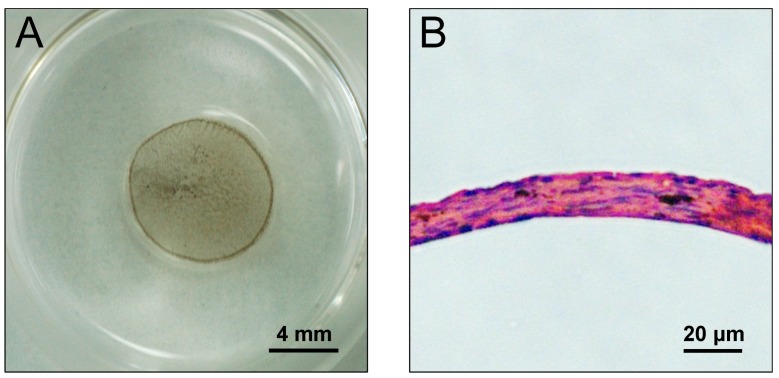
Photographs of a DEC sheet created by the Mag-TE system. DECs labeled with MNPs were seeded into a 24-well ultra low attachment culture plate (Corning Inc., Corning, NY, USA) at 2 × 10^6^ cells/plate. A cylindrical neodymium magnet (magnetic force, 4110 G) was placed on the reverse side of the ultra low attachment plate, and the cells were cultured for 24 h. After the culture, the cell sheet was constructed (**A**). Microscopic image of an HE-stained cross section of a DEC sheet (×100) (**B**).

We attempted to construct DEC and DMC sheets using the Mag-TE system [14]. By applying magnetic force, both DECs and DMCs labeled with MNPs migrated to form a sheet-like structure in the middle of the culture well after 24 h of incubation (Figure 5A). The cell sheets were circular with diameters of 8 mm and appeared to have the same structure in repeated experiments. These sheets were embedded in paraffin, sectioned, and observed after hematoxylin and eosin (HE) staining. The cell sheets had a multilayered structure of approximately 20 μm in thickness (Figure 5B).

### 2.6. Localization of MNPs in CC Sheet

**Figure 6:**
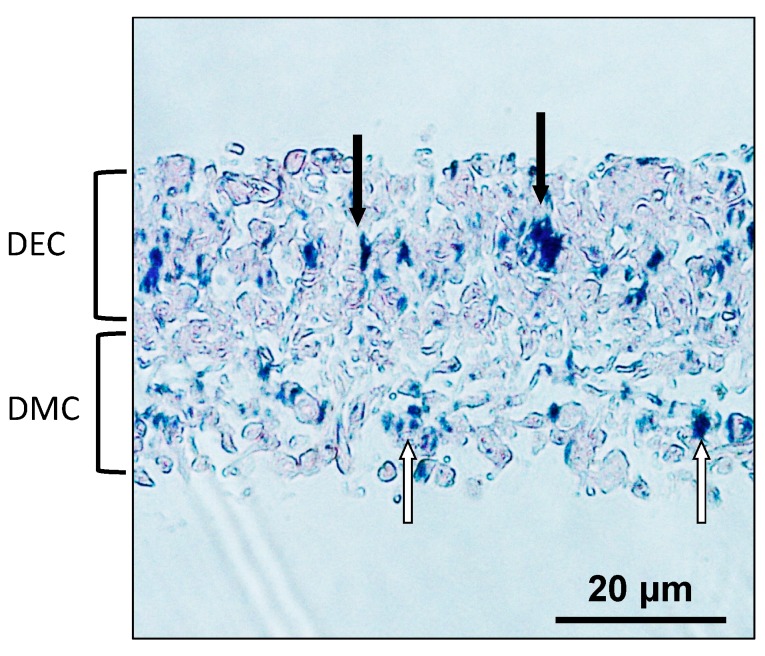
Microscopic observation of a CC sheet after iron staining (×200). Filled arrows indicate MNPs in the DEC layer. White arrows indicate MNPs in the DMC layer. Scale bar = 20 μm.

A CC sheet was constructed using the Mag-TE system. The CC sheet had a multilayered structure approximately 40 μm in thickness. Iron staining was performed to visualize the localization of MNPs in CC sheet. MNPs were distributed ubiquitously over the CC sheet (Figure 6).

### 2.7. Expression of mRNAs Encoding Enamel Matrix- and Dentin-Related Genes in CC Sheet

**Figure 7:**
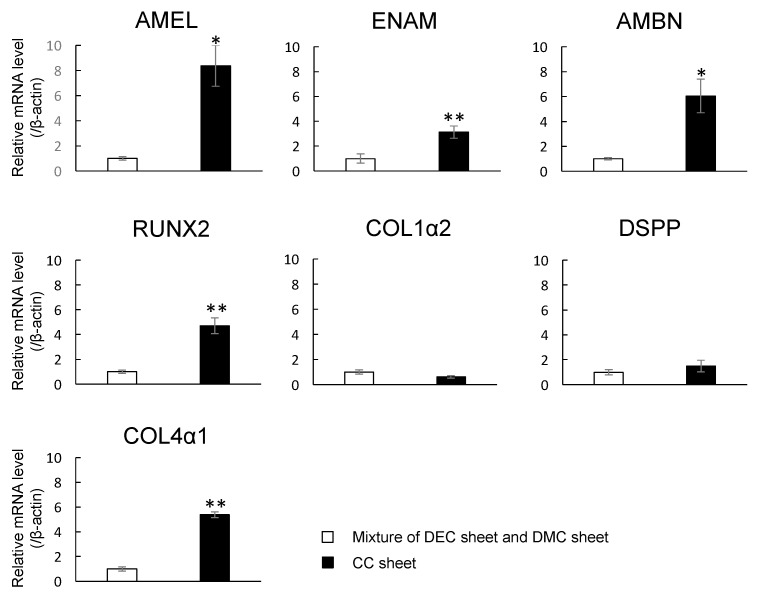
mRNA expression levels of *AMEL*, *ENAM*, *AMBN*, *RUNX2*, *COL1α2*, *DSPP*, and *COL4α1* in CC sheet examined by real-time RT-PCR. A mixture of DEC and DMC sheets prepared separately were used as a control. Real-time RT-PCR data were normalized to the expression levels of *β-actin* mRNA. Independent experiments were repeated twice. Data represent the mean ± SD of triplicate samples. * *p* < 0.05. ** *p* < 0.01.

The mRNA expression levels of *AMEL*, *ENAM*, *AMBN*, *RUNX2*, and *Collagen IV 1* (*COL4 1*) in CC sheet were higher than those in the control (* *p* < 0.05, ** *p* < 0.01), although there were no significant changes in the levels of *COL1α2* or *DSPP* mRNA expression (*p* > 0.05) (Figure 7).

### 2.8. Localization of Collagen IV in CC Sheet

**Figure 8:**
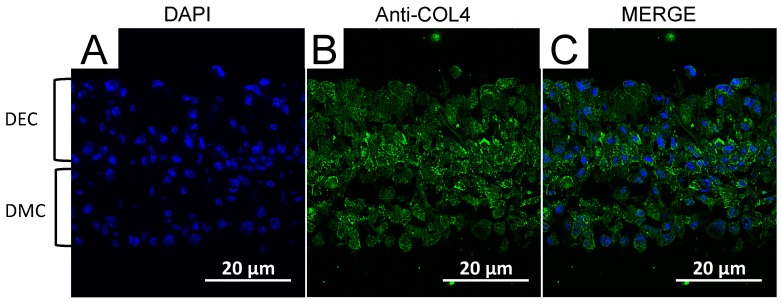
Fluorescence microscopic observation of a CC sheet (×200). DAPI (blue) was used to stain nuclei (**A**). COL4 was stained with a specific antibody. COL4-expressing cells (green) were identified around the border of DEC and DMC layers (**B**,**C**). Scale bars = 20 μm.

Immunofluorescence staining was performed to assess the localization of Collagen IV(COL4) that is expressed in the basement membrane of presecretory and late mature stage tooth germs. The nuclei of live cells were stained with 4′,6-diamidino-2-phenylindole (DAPI) (Figure 8A). COL4 expression was localized markedly in the middle region of the CC sheet (Figure 8B,C). 

## 3. Discussion

Recently, MNPs have been attracting a lot of attention in medical field. Due to their special and unique properties, they have been studied and applied clinically in various medical areas, including regeneration. In the field of regeneration, tooth regeneration using tissue engineering techniques has been studied and developed actively, but the techniques or methods are not well established [19]. Tooth development is regulated by intricate interactions between DECs and DMCs, but it has not been completely elucidated yet [15,17]. In particular, DE is formed through a complicated development process [20]. Therefore, many problems remain in the field of enamel regeneration. For example, although a collagen sponge has been used as a scaffold to transplant a mixture of DECs and DMCs, the success rate of tooth production is not very high [21]. This result indicates that it is essential for enamel regeneration to control the three-dimensional position of DECs and DMCs to be similar to the tooth developmental process. Although DECs and DMCs are aligned and in contact via the basement membrane throughout tooth development [22], it is difficult to mimic this microenvironment in vitro. Therefore, we focused on a cell sheet and determined whether we could reproduce the cell arrangement and three-dimensional cell position in vitro by layering two cell sheets of two different cell types. MNPs have an ability to make different cells cohere together by applying magnetic force [12]. This is the reason why we adopted the method employing MNPs among the various methods used to prepare cell sheets, such as using temperature-responsive cell culture dishes [23,24] and collagen gels [25]. 

Although MNPs are applied to regenerative medicine of many organs and are known as a biocompatible material [10,11,12,13,14,26], neither the influence of MNPs on odontogenic cells nor their applicability to regenerative dentistry have been reported. First, we examined the internalization of MNPs in the odontogenic cells by TEM. The TEM imaging revealed that MNPs were localized in the cytoplasm, and it is suggested that MNPs were taken up in the odontogenic cells (Figure 1). This result was similar to previous research which reported the uptake of MNPs in different cell types [27,28,29]. The result obtained in the present study and the previous reports [27,28,29] suggest that MNPs can be used as carriers to deliver biologically active agents, such as growth factors and nanovectors, to odontogenic cells for control of histodifferentiation and organ generation in vivo [30,31]. We further investigated the cytotoxicity of MNPs in DECs and DMCs. The results of MTS assays suggested that MNPs had no cytotoxicity in DECs or DMCs up to 300 pg-magnetite/cell (Figure 2). Therefore, we believed that we could apply MNPs to DE regeneration. In this study, a DEC sheet, DMC sheet, and combined cell sheet of DECs and DMCs (CC sheet) were constructed using the Mag-TE system. The CC sheet was constructed by directly laminating magnetically labeled DECs on a prepared DMC sheet by magnetic force loading. Although cell sheets can be piled up by a method involving temperature-responsive cell culture dishes, the cell sheets prepared in this way must be peeled off and placed on another cell sheet. As cell sheets are thin, they require skill in handling to multi-layer sheets one by one [23,24,32,33]. Therefore, the multi-layering technique with the Mag-TE system will be useful because the manipulation required to remove a cell sheet can be avoided. Furthermore, in the method with temperature-responsive cell culture dishes, it is difficult to cohere two cell sheets together [34], whereas in our method with the Mag-TE system, layers of DECs and DMCs could be brought into close proximity by magnetic force. It is suggested that epithelial–mesenchymal interaction between the DEC layer and DMC layer is more likely to occur when the Mag-TE system is applied. In this CC sheet, the mRNA levels of *AMEL*, *ENAM*, *AMBN*, *RUNX2*, and *COL4α1* were significantly higher than those in a mixture of DEC and DMC sheets prepared separately. *AMEL*, *ENAM*, and *AMBN* are differentiation markers of DECs which are known to differentiate into ameloblasts [35,36,37,38,39,40,41]. Therefore, our results suggest that DECs differentiated into enamel-secreting ameloblasts [42], and imply that epithelial–mesenchymal interactions proceeded by bringing DEC and DMC layers physically close to each other by magnetic force. *RUNX2* is a differentiation marker of osteoblasts and odontoblasts [43]. The increase in *RUNX2* mRNA in the CC sheet suggests that DMCs differentiated into odontoblasts because of the enhancement of epithelial–mesenchymal interactions. The result of the iron staining (Figure 6) indicated that the MNPs were distributed ubiquitously over the CC sheet. This suggests that the magnetic force can be loaded uniformly to the CC sheet, and that epithelial–mesenchymal interactions may occur homogeneously in the CC sheet. The basement membrane, which interfaces with the dental epithelium and papilla mesenchyme [44,45], is involved in epithelial–mesenchymal interactions [46,47]. COL4 is a membrane form of collagen expressed in tooth bud basement membrane at the presecretory stage and reported to be related to ameloblast differentiation and tooth development [39,45,48,49]. These studies suggest that the interactions mediated through the basement membrane and the basement membrane components such as COL4 are essential for DEC growth and differentiation to form the proper shape and size of the tooth. Taken together with these previous findings, the results in the present study, which showed enhancement of *COL4α1* mRNA expression and localization of COL4 by immunofluorescence staining in CC sheet, suggest the existence of a basement membrane between the DEC and DMC layers (Figure 8B,C). 

Our results collectively support the hypothesis that epithelial–mesenchymal interactions between DEC and DMC layers were induced by the Mag-TE system. Further study is required to verify the utility of CC sheets employing MNPs as a cutting edge technology, including tissue regenerating experiments in vivo. 

## 4. Materials and Methods 

### 4.1. Materials

MNPs (Nano3D Biosciences, Houston, TX, USA) and a neodymium magnet (diameter = 25 mm, magnetic force = 4110 G; NeoMag Co., Ltd., Chiba, Japan) were used in this study. MNPs (0.05%, *w*/*v*) coated with poly-l-lysine were diluted to 0.001% (*w*/*v*) with Dulbecco’s modified Eagle’s medium (DMEM; Nacalai Tesque Inc., Kyoto, Japan) containing 10% fetal bovine serum (FBS; Hyclone^®^; Thermo Fisher Scientific Inc., Waltham, MA, USA) and 1% antibiotics (Gibco^®^ Antibiotic-Antimycotic; Thermo Fisher Scientific Inc., Waltham, MA, USA). 

### 4.2. Primary Culture of Odontogenic Cells In Vitro

DECs and DMCs were isolated from third molar tooth buds of 6-month-old porcine lower jaws (Fukuokashokunikuhanbai Co. Ltd., Fukuoka, Japan) and cultured as described previously [16]. Single cell suspensions of DECs and DMCs were seeded in 10-cm dishes (Corning Inc., Corning, NY, USA) and maintained in DMEM containing 10% FBS and 1% antibiotics at 37 °C with 5% CO_2_. 

### 4.3. Transmission Electron Microscopy (TEM)

Odontogenic cells were incubated with MNPs (100 pg-magnetite/cell) for 24 h. After incubation, cells were collected, washed three times with phosphate buffered saline (PBS), and fixed with a fix buffer (2.5% glutaraldehyde, 0.1 M sucrose, 3 mM CaCl_2_, and 0.1 M sodium cacodylate, pH 7.4) for 2 h. Next, the cells were rinsed in 0.1 M sodium phosphate for 15 min at room temperature. The cells were then postfixed with 1% OsO_4_ for 1.5 h and rinsed in 0.1 M sodium phosphate overnight at 4 °C. Subsequently, the cells were dehydrated in graded alcohol concentrations and propylene oxide, embedded in epoxy resin, and incubated for 2 days at 65 °C. Ultrathin sections (80 nm) were prepared by a Leica EM UC7 (Leica microsystems GmbH, Wetzlar, Germany). Finally, the sections were stained with 2% uranyl acetate and lead acetate for 5 and 10 min, respectively. The sections were observed using a Tecnai-20 (FEI Co., Hillsboro, OR, USA).

### 4.4. Cytotoxicity Assay

The cytotoxicity of MNPs in DECs and DMCs was assessed by MTS assays. In brief, DECs or DMCs were seeded onto a 96-well flat bottom cell culture plate (Corning Inc., Corning, NY, USA) at 3 × 10^3^ cells/well and incubated with MNPs at 0, 50, 100, 150, or 300 pg-magnetite/cell. After 24 h of incubation at 37 °C, the cells were exposed to an MTS solution for 24 h. Suspended cells were removed by gentle rinsing with phosphate-buffered saline (PBS), and the number of adherent cells remaining in each well was quantified using a coupled enzymatic assay resulting in conversion of a tetrazolium salt into a red formazan product (CellTiter 96 Aqueous Non-Radioactive Cell Proliferation Assay, Promega, Madison, WI, USA). Recording the absorbance at 490 nm in the MTS assay was carried out using a microplate reader (infinite M200, Tecan Japan Co., Ltd., Kanagawa, Japan).

### 4.5. Construction of Cell Sheets

DEC and DMC sheets were constructed using the Mag-TE system [10,14,26]. To magnetically label the cells, DECs and DMCs were first incubated with MNPs at 100 pg-magnetite/cell. After 6 h of incubation at 37 °C, both cell types labeled with MNPs were seeded into a 24-well ultra low attachment cell culture plate (Corning Inc., Corning, NY, USA) at 2 × 10^6^ cells/plate. The cylindrical neodymium magnet (4110 G) was placed on the reverse side of the ultra low attachment plate, and the cells were cultured for an additional 24 h. The external magnetic field was provided perpendicular to the cell layers. Then, the neodymium magnet was removed from the culture plate. A CC sheet was also constructed using the Mag-TE system. First, 2 × 10^6^ magnetically labeled DMCs were seeded into a 24-well ultra low attachment cell culture plate with the external magnetic field provided perpendicular to the cell layers and cultured for 24 h. After generation of the DMC sheet, 2 × 10^6^ magnetically labeled DECs were seeded into the same 24-well ultra low attachment cell culture plate and incubated for another 24 h. Then, the neodymium magnet was removed from the culture plate.

### 4.6. Real-Time Reverse Transcriptase Polymerase Chain Reaction (RT-PCR) Analyses

Total RNA was extracted using TRIzol reagent (Invitrogen, Carlsbad, CA, USA). First-strand cDNA was synthesized from 100 ng total RNA using ReverTra Ace (Toyobo, Osaka, Japan). The cDNA was then amplified by SYBR green 1 DNA polymerase (TAKARA BIO Inc., Shiga, Japan). Real-time RT-PCR analyses of *Amelogenin* (*AMEL*) (Sigma-Aldrich Co. LLC., Tokyo, Japan), *Enamelin* (*ENAM*) (Sigma-Aldrich Co. LLC., Tokyo, Japan), *Ameloblastin* (*AMBN*) (Sigma-Aldrich Co. LLC., Tokyo, Japan), *Runt-related transcription factor 2* (*RUNX2*) (Sigma-Aldrich Co. LLC., Tokyo, Japan), *CollagenIα2* (*COL1α2*) (Sigma-Aldrich Co. LLC., Tokyo, Japan), *Dentin sialophosphoprotein* (*DSPP*) (Sigma-Aldrich Co. LLC., Tokyo, Japan), *CollagenIVα1* (*COL4α1*) (BEX CO., LTD., Tokyo, Japan), and *β-actin* (Sigma-Aldrich Co. LLC., Tokyo, Japan) were performed using a Rotor-Gene 6000 (Qiagen, Tokyo, Japan). *β-Actin* was chosen as an internal control to standardize the variability in amplification owing to slight differences in total starting RNA concentrations. Primer and probe sequences are listed in Supplementary Table S1. 

### 4.7. Prussian Blue Staining

To visualize the localization of MNPs in CC sheet, an iron staining kit (Muto pure chemicals Co., Ltd., Tokyo, Japan) was used. A CC sheet constructed by the Mag-TE system was fixed with 4% paraformaldehyde and then rinsed with PBS three times. After dehydrating in graded alcohol concentrations, the samples were embedded in paraffin, and 3 μm-thick serial sections were prepared and mounted on glass slides. Deparaffinized sections were stained with the mixture of filtrated 2% potassium ferrocyanide II/2% hydrochloric acid (ratio 1:1) for 60 min at 37 °C and washed in distilled water. Finally, the sections were counter-stained with 1% safranin O for 5 min, washed three times in distilled water, and air dried. 

### 4.8. Immunohistochemical Observations

A CC sheet constructed by the Mag-TE system was fixed with 4% paraformaldehyde and then rinsed with PBS three times. After dehydrating in graded alcohol concentrations, the samples were embedded in paraffin, and 3 μm-thick serial sections were prepared and mounted on glass slides. Deparaffinized sections were incubated in an antigen retrieval solution (HistoVT One, Nacalai Tesque, Kyoto, Japan) for 20 min at 90 °C, followed by blocking with EzBlock BSA (ATTO, Tokyo, Japan) for 30 min. The sections were incubated overnight with a primary antibody against COL4 (anti-collagen IV antibody, ab6586, Abcam Co., Ltd., Tokyo, Japan), which is a basement membrane marker, followed by 30 min of incubation with *Alexa Fluor 488*-conjugated secondary donkey anti-rabbit IgG (Thermo Fisher Scientific Inc., Waltham, MA, USA). Then, the sections were gently rinsed three times with PBS and counterstained with 4′,6-diamidino-2-phenylindole (DAPI) (Dojindo, Kumamoto, Japan) for 10 min. Fluorescence images were acquired with a BZ-9000 (Keyence, Osaka, Japan).

### 4.9. Statistical Analyses

Differences between group averages were assessed by Student’s *t*-tests or one-way analysis of variance (ANOVA) with Tukey’s multiple range test. SPSS version 20.0.0 (IBM SPSS Statistics, IBM, Tokyo, Japan) was used for statistical analyses.

## 5. Conclusions

In the present study, MNPs were shown to be taken up in odontogenic cells, and were found to be a biocompatible material for odontogenic cells at a limited concentration. The Mag-TE system demonstrated that the epithelial–mesenchymal interactions and the differentiation stage of DECs could be controlled, which is thought to be important to regenerate DE. Regeneration of DE can be expected in vivo by reconstructing the microenvironment and differentiation stage of odontogenic cells in vitro. Consequently, MNPs may be a promising and unique material for regenerative dentistry.

## Figures and Tables

**Table 1:** Classification of experimental groups depending on the presence of MNPs or magnetic force loading.

Classification of The Experimental Groups	Group 1	Group 2	Group 3	Group 4
MNPs	-	-	+	+
Magnetic Force	-	+	-	+

## Supplementary Materials

The following are available online at www.mdpi.com/2079-4991/7/10/322/s1, Table S1: Primers/probes for real-time RT-PCR.
