# Supplementary material for: Porcine Dental Epithelial Cells Differentiated in a Cell Sheet Constructed by Magnetic Nanotechnology
Source: Nanomaterials (Basel). 2017 Oct 13;7(10):322. doi: 10.3390/nano7100322 (PMC5666487; doi:10.3390/nano7100322)
Supplement: Supplementary file 1 [file nanomaterials-07-00322-s001.pdf]

Supplementary Table 1. Primers used for real-time RT-PCR

Target name	Primer and probe	Sequence (5'-3')
AMEL	Primer F	ACCTGGATTTTGTTTGCTTGCC
	Primer R	AGCACCTCATAGCTGAAGTTGATA
ENAM	Primer F	TCCCAAAACAGAGACTCCAGC
	Primer R	GGTTCCTGTTGGGCTGGTG
AMBN	Primer F	AGGTGGCACCATCAGAAAAG
	Primer R	TCAAACGGGCTATTGGAAAC
RUNX2	Primer F	GTGTGAATGCTTCATTCGCCTC
	Primer R	GTCTCGGTGGCTGGTAGTGA
COL1a2	Primer F	GGTCACAACGGTCTGGATGG
	Primer R	CGACACGTCCTCTCTCACCA
DSPP	Primer F	AAGCGCCAGTACAGGATGAG
	Primer R	TGCTGCCTTCCTATGTCATGG
COL4a1	Primer F	CCCCAAGTAGGCAGAAATGA
	Primer R	ACGAAAGCTTGACCTGCCTA
β-actin	Primer F	CCACACTGTGCCCATCTACG
	Primer R	GTGGTGGTGAAGCTGTAGCC

Note. Forward primers (Primer F) and reverse primers (Primer R) are listed.
